# Development of a field-deployable RPA-CRISPR/Cas12a assay for the detection of *Cyclospora cayetanensis* in human feces

**DOI:** 10.1186/s13071-025-07150-x

**Published:** 2025-11-28

**Authors:** Ziyang Qin, Yilin Wang, Mengqing Sun, Qinglin Wang, Junxia Duan, Chunhao Gu, Xinfu Zhang, Fuchang Yu, Yayun Wu, Huiyan Xu, Junqiang Li, Longxian Zhang

**Affiliations:** 1 College of Veterinary Medicine, Henan Agricultural University, No. 218 of Ping An Avenue, Zhengdong Newly-Developed Area, Zhengzhou, 450046 People’s Republic of China; 2 International Joint Research Laboratory for Zoonotic Diseases of Henan, Zhengzhou, 450046 Henan Province China; 3 Key Laboratory of Quality and Safety Control of Poultry Products, Ministry of Agriculture and Rural Affairs, Beijing, People’s Republic of China; 4 Gene Editing Center for Veterinary Medicine, Henan Agricultural University, Zhengzhou, 450046 China; 5 College of Animal Science and Technology, Tarim University, Alar, Xinjiang 843300 People’s Republic of China

**Keywords:** *Cyclospora cayetanensis*, Recombinase polymerase amplification, CRISPR/Cas12a, Visualized detection, On-site detection

## Abstract

**Background:**

*Cyclospora* is an emerging intestinal pathogenic protozoan transmitted through foodborne and waterborne routes. At least 19 countries in the world have recorded outbreaks of cyclosporiasis, mainly associated with the consumption of contaminated fresh agricultural products. The lack of a sensitive immediate test is one of the major obstacles to the rapid diagnosis of cyclosporiasis. The target interference mechanisms of clustered regularly interspaced short palindromic repeats (CRISPR) and CRISPR-associated (Cas) protein systems have been adapted into versatile and efficient genome manipulation and disease-curing technologies, while also being promising for point-of-care testing (POCT) applications. It can serve as an excellent rapid and specific detection tool.

**Methods:**

The recombinase polymerase amplification (RPA) and the CRISPR/Cas12a system were combined to develop a detection method for *C. cayetanensis* (termed RECCT-Cay) via visual observation of fluorescent readings under blue light and field diagnosis using lateral flow strip (LFS) biosensors.

**Results:**

The detection limit of the established RECCT-Cay was 7 copies/μL. Under simulated clinical conditions, the detection limit was 30 oocysts per gram of stool. At the same time, the established detection platform can distinguish *C. cayetanensis* from the closely related *Eimeria* spp. The results of our constructed assay were compared with nested PCR, and the detection results of 30 clinical stool samples were consistent, with three samples positive for *C. cayetanensis*. Based on the RECCT-Cay detection principle, a portable suitcase-sized device has been designed, which can conduct rapid on-site detection of clinical samples.

**Conclusions:**

The RECCT-Cay platform features rapid speed, high sensitivity, and the capability for field detection, making it a promising tool for use in remote areas.

**Graphical Abstract:**

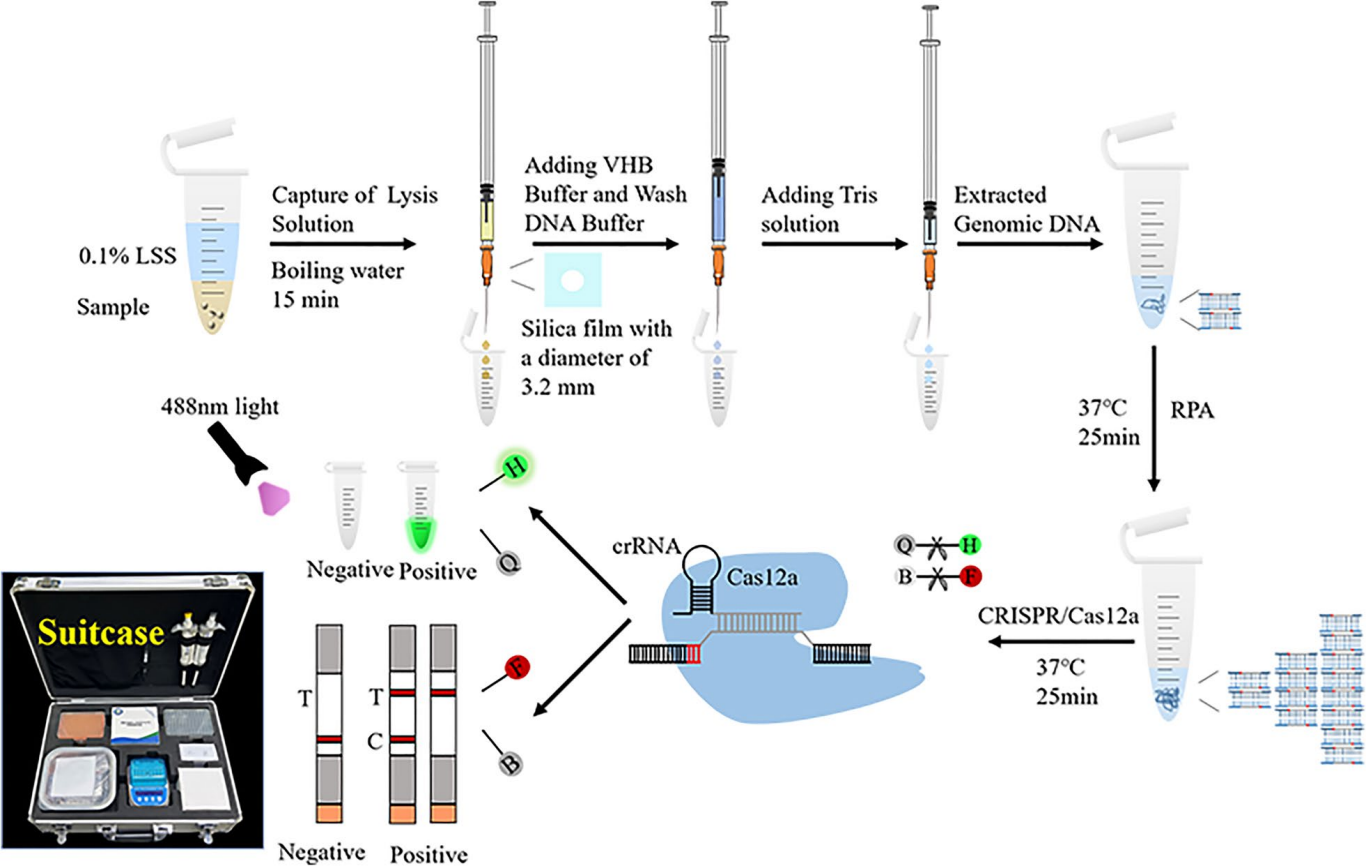

**Supplementary Information:**

The online version contains supplementary material available at 10.1186/s13071-025-07150-x.

## Background

*Cyclospora* is an emerging intestinal pathogenic protozoan transmitted through foodborne and waterborne routes [1–3]. *Cyclospora cayetanensis* is the only known species of *Cyclospora* that infects humans [4]. However, there is a suggestion that, based on differences in the genome of *C. cayetanensis*, it is divided into three species that cause human cyclosporiasis, and this view has not yet been widely accepted [5].

*C. cayetanensis* has emerged as a significant public health and food safety concern[6], particularly in developed countries, where it is frequently linked to foodborne outbreaks of intestinal diseases associated with the consumption of contaminated fresh produce [7]. Notably, beyond its role in foodborne outbreaks in these regions, it is also an important cause of diarrhea in children under 4 years of age in endemic countries [8]. *C. cayetanensis* not only causes diarrhea in the host, but also leads to abdominal pain, nausea, vomiting and other gastrointestinal symptoms. Moreover, it may cause persistent diarrhea or even death in immunocompromised individuals [9].

Since 2010, *C. cayetanensis* has been reported in 54 countries. Among them, 19 countries have reported a total of 8597 outbreaks, with the USA and Canada accounting for the largest number of cases [10]. In view of this, the U.S. Food and Drug Administration (FDA) issued the “Action Plan for *C. cayetanensis* Prevention, Response, and Research” in 2021, aiming to reduce the harm to public health caused by foodborne cyclosporiasis. Therefore, point-of-care testing (POCT) technologies, which refer to rapid, reliable, and sensitive detection methods using portable instruments, have become increasingly important for the detection of *C. cayetanensis*.

At present, morphological microscopy is a commonly used method for *C. cayetanensis* detection, which required large workload, experienced laboratorians, expensive equipment, is time-consuming, and lacks specificity and sensitivity [11, 12]. The FDA recommends the use of molecular biology techniques, such as real-time PCR and nested-PCR, for the detection of *C. cayetanensis*. Although PCR detection is relatively faster and cheaper than traditional microbiological testing, it still requires professional laboratories, experimental instruments and skilled technicians, and is not suitable for POCT. Currently, isothermal amplification methods, such as loop-mediated isothermal amplification (LAMP) and recombinase polymerase amplification (RPA), operate under isothermal conditions, making them most suitable for on-site testing [13].

The clustered regularly interspaced short palindromic repeats (CRISPR)/CRISPR-associated (CRISPR/Cas) systems are adaptive antiviral immune mechanisms found in most archaea and many bacteria [14–16]. During their target interference process, a guiding RNA specific to the CRISPR/Cas systems recognizes invading nucleic acids by base-pairing and activates Cas proteins or protein complexes to hydrolyze these nucleic acids [17–19]. The target interference mechanisms of CRISPR/Cas systems have been adapted into versatile and efficient genome manipulation and disease-curing technologies [20, 21], while also being promising for POCT applications [22]. Chen et al. combined the nonspecific cleaving ability of Cas12a with the nucleic acid amplification ability of recombinase polymerase amplification (RPA) to develop a DNA detection platform termed DETECTR (DNA endonuclease targeted CRISPR trans reporter) [18]. In the DETECTR detection platform, the target dsDNA is first amplified by RPA. Cas12a is then guided to the dsDNA target by complementary crRNA, forming a Cas12a–crRNA–dsDNA terpolymer complex. At this time, Cas12a trans-cleaves the ssDNA reporter molecule that carries a fluorophore and quencher, resulting in the separation of the quencher and fluorophore, thereby releasing the fluorescence signal that indicates the presence of the target DNA [18]. The DETECTR detection platform represents a good rapid and specific detection method. The RPA reaction can be carried out at body temperature, which is conducive to its application in remote areas [23, 24]. In this study, to develop a rapid, reliable, sensitive, and specific POCT method for *C. cayetanensis*, we developed a detection method using the recombinase polymerase amplification (RPA) and CRISPR/Cas12a system (termed RECCT-Cay). The detection results can be observed by the naked eye under blue light or through a lateral flow strip (LFS). We also designed and developed a portable suitcase, which includes minimal equipment (e.g., pipettes, thermostatic metal baths, and lateral flow strips) and reagents (e.g., lyophilized RPA and CRISPR/Cas12a reagents). The portable suitcase is designed for ease of storage and transport, providing a “one-stop shop” for sample collection, rapid fecal DNA extraction, and the RECCT-Cay. This method is simple to operate and is suitable to use in clinics, remote areas, and other locations for preliminary screening and diagnosis of *C. cayetanensis*, while also providing a technical reference for future POCT developments.

## Methods

### Reagents and oligonucleotides

All primers, quenched fluorescent DNA reporter (HEX-12N-BHQ1), and lateral flow strip test reporter (FAM-12N-Biotin) were obtained from Sangon Biotech (Zhengzhou, China). All the nucleotide sequences are listed in Additional file 1: Supplementary Table S1. Annealing buffer for DNA Oligos (5 ×) was purchased from Beyotime Biotechnology (Shanghai, China); HiScribe™ T7 High Yield RNA Synthesis Kits and 10 × NE Buffer 2.1 were purchased from New England Biolabs (Ipswich, MA, USA); Recombinant DNase I (RNase free) and RNase inhibitor were purchased from TaKaRa Bio Inc. (Dalian, China); the TwistAmp® Basic kit was purchased from TwistDx Ltd. (Hertfordshire, UK); recombinant *Francisella novicida* Cas12a (FnCas12a) protein and *Lachnospiraceae bacterium* (LbCas12a) were purchased from Tolo Biotech (Shanghai, China); the lateral flow strip biosensor (JY0301) was purchased from Warbio Biotech (Nanjing, China); the TIANamp Stool DNA Kit and Universal DNA Purification Kit were purchased from TIANGEN (Beijing, China); and the other regents used in this study were purchased from Solarbio Science and Technology Co., Ltd. (Beijing, China).

### Sample information

The reference isolate of *C. cayetanensis* used as the positive control was sourced from our laboratory. Its strain designation is HNKFCc2012-1203 and it was isolated from a clinical stool sample of a confirmed cyclosporiasis male patient in Kaifeng city, Henan Province, China, in 2012. The DNA of *Eimeria tenella*, *Eimeria cylindrica*, *Toxoplasma gondii*, *Cryptosporidium parvum*, *Giardia duodenalis*, *Blastocystis* sp., and *Enterocytozoon bieneusi* was stored in our laboratory (Detailed information in Additional file 1: Supplementary Table S2). A total of 30 human fecal samples were collected from a hospital in Kaifeng city, Henan Province, China, in 2023, and stored in our laboratory (Detailed information in Additional file 1: Supplementary Table S3).

### Extraction of fecal genomic DNA

For the commercial kit extraction method, according to the manufacturer’s instructions, fecal genomic DNA was extracted using the TIANamp Stool DNA Kit (TIANGEN, China). The DNA quality was assessed using NanoDrop One (Thermo Fisher Scientific Inc., Waltham, MA, USA).

For the surfactant pyrolysis extraction method, following previous studies [25], the *C. cayetanensis* oocysts were lysed with 0.1% N-lauroylsarcosine sodium salt (LSS) to release their DNA, and a 1-mL sterile syringe (with a silica gel membrane for nucleic acid purification) was used instead of a nucleic acid-purified DNA column (Additional file 1: Supplementary Fig. S1). The *C. cayetanensis* oocyst solution was added to 0.1% LSS solution and incubated in boiling water for 5 min. The heating was then stopped, and the mixture was incubated for an additional 10 min using the residual heat of the boiling water. The lysate was transferred to a homemade DNA banding column with a 1-mL syringe and pushed into the column under syringe pressure. Next, 600 μL of VHB Buffer and 600 μL of DNA Wash Buffer (E.Z.N.A.® Stool DNA Kit, OMEGA, USA) were sequentially added to purify the DNA banding columns. Finally, 50 μl of 10 mM Tris solution was added to the column. After incubation at room temperature for 3 min, DNA was eluted with Tris under syringe pressure.

### Design and preparation of RPA primers and crRNA

The cytochrome b (*Cytb*) gene and the cytochrome c oxidase subunit 3 (*Cox*3) gene (GenBank accession No. KP658101.1) of *C. cayetanensis* genomic DNA were chosen as the amplification targets. RPA primers were designed using the Prime-BLAST online tool, with forward and reverse primers ranging from 28–35 nucleotide (nt) (Additional file 1: Supplementary Table S1). Then, one crRNA was screened for each gene. To identify highly conserved regions specific to *C. cayetanensis* specificity, different *C. cayetanensis* sequences were downloaded from GenBank and aligned using MEGA 7.0 (https://www.megasoftware.net/). Then, we selected a 21–24 nt sequence followed by a T-nucleotide-rich PAM as the target sequence. The sscrDNA-F sequence, which included the T7 promoter, and the sscrDNA-R sequence, which was the complement of the sscrDNA-F sequence, were synthesized (Additional file 1: Supplementary Table S1). The crRNA preparation was performed as follows: (1) annealing: the synthesized sscrDNA-F and sscrDNA-R were annealed to form dscrDNA; (2) transcription: dscrDNA was transcribed into crRNA using the HiScribe™T7 High Yield RNA Synthesis Kit at 37°C for 16 h; and (3) purification: the NucAway™Spin Column was used to purify crRNA from the previously transcribed product. The concentration of the purified crRNA was measured using a NanoDrop One instrument (Additional file 1: Supplementary Table S3). Then, annealing products, transcription products, and purified crRNA were subjected to nondenatured RNA gel electrophoresis (Additional file 1: Supplementary Fig. S2).

### Recombinase polymerase amplification (RPA) assay

RPA amplification was performed using the TwistAmp® Basic kit according to the manufacturer’s instructions. Briefly, the TwistAmpexo enzymes were dissolved in a mixture consisting of 29.5 μL of supplied rehydration buffer, 2.4 μL of forward primer (10 μM), 2.4 μL of reverse primer (10 μM), 8.2 μL of sterile nuclease-free water, and 5 μL of DNA template. After the TwistAmpexo enzymes were dissolved in the mixture, 2.5 μL of 280 nM MgOAc was added to the inner surface of the tube lid, and the lid of the reaction tube was carefully closed. The solution was briefly rotated to mix the MgOAc, which triggered the RPA reaction. The reaction was performed at 37 °C for 25 min.

### Fluorescence detection on the RECCT-Cay platform

For RECCT-Cay platform fluorescence detection, the 20 μL reaction system was prepared, which included 2 μL of 10 × NE Buffer 2.1, 1 μL of RNase inhibitor (40 U), 1 μL of HEX-12N-BHQ1 reporter (20 μM), 1 μL of LbCas12a (50 μM), 1 μL of purified crRNA (1 μM), 2 μL of target DNA (unpurified RPA products), and 12 μL of sterile nuclease-free water. The real-time fluorescence values in the HEX channel were measured using a qTower 3G qPCR system (Analytik Jena, Germany) every 30 s for a total of 25 min, and the reaction temperature was 37 °C.

### LFS experiment on the RECCT-Cay platform

To be able to diagnose *C. cayetanensis* in the field, we use LFS assays, which allow the results to be read without the need for complex instruments and skilled technicians. A colloidal gold-labeled anti-FAM antibody was fixed at the lower end of the binding pad, which could bind to the FAM antigen at the 5’ end of the F-B reporter to form a complex. According to the CRISPR-Cas12 system’s mechanism, when the F-B reporter is not degraded by LbCas12a, the complex is captured by biotin ligands fixed to the control line (C-line). When the F-B reporter was degraded by LbCas12a, it could not bind all the gold-labeled antibodies, and some of the free gold nanoparticles were captured by the antibodies on the test lines (T-line).

The 20 μL LFS assay system consisted of 2 μL of 10 × NE Buffer 2.1, 1 μL of RNase inhibitor (40 U), 10 μL of HEX-12N-BHQ1 reporter (20 μM), 1 μL of LbCas12a (50 μM), 1 μL of purified crRNA (1 μM), 2 μL of target DNA (unpurified RPA products), and 3 μL of sterile nuclease-free water. The reaction was conducted in a constant temperature incubator at 37 °C for 25 min, followed by the addition of sterile nuclease-free water to bring the total volume to 50 μL. The LFS pad was then placed in a 50 μL RPA-CRISPR/Cas12a mixture and allowed to react at room temperature for 5–7 min. Finally, results were interpreted according to the presence of a test line (T-line).

### The specificity and sensitivity of the RECCT-Cay platform

First, the sensitivity of nucleic acid detection was evaluated via PCR amplification of *C. cayetanensis* targeting the *Cox*3 gene locus (primer sequences are listed in Additional file 1: Supplementary Table S1) [26]. Prior to gel block recovery of the target fragments, the PCR amplification products were subjected to bidirectional sequencing by Sangon Biotech (Zhengzhou, China) to confirm the correctness of the amplified target sequence, ensuring that the obtained fragments matched the expected *Cox*3 gene region of *C. cayetanensis*. Subsequently, gel blocks containing the target fragments were recovered (Additional file 1: Supplementary Fig. S3), and the target DNA fragments were purified using a Universal DNA Purification Kit (TIANGEN, China). The concentration of the purified fragments was then measured with a NanoDrop One. Tenfold serial dilution of the above DNA was analyzed to determine the sensitivity of the developed RECCT-Cay platform. Second, to evaluate oocyst sensitivity, *C. cayetanensis* oocysts were purified on the basis of previous research [27], including raw fecal filtration, sucrose gradient centrifugation, and cesium chloride gradient centrifugation. The resulting solution was stored in phosphate buffer solution (PBS) at 4 °C. Purified *C. cayetanensis* oocysts were counted using hemocytometry. A certain volume of the purified oocyst suspension was then inoculated into a negative fecal sample to achieve a concentration of 3 × 10^5^ oocysts per gram (OPG), followed by genomic DNA extraction. A tenfold serial dilution of this DNA was analyzed to further assess the sensitivity of the RECCT-Cay platform.

To ensure the method specifically detects *C. cayetanensis* without cross-reactivity with phylogenetically related or clinically relevant protozoa, the specificity assay included parasites that either are closely related coccidia (*E. tenella*, *E. cylindrica*), common human intestinal protozoa (*C. parvum*, *G. duodenalis*, *Blastocystis* sp., and *E. bieneusi*), or a zoonotic protozoan with distinct host-specific intestinal stages (*T. gondii*, whose sexual reproduction is restricted to felids, not humans).

### RECCT-Cay for clinical sample detection

Fecal samples were collected from 30 patients at a hospital in Henan province. To evaluate the suitability of our constructed RECCT-Cay platform, DNA from these 30 fecal samples was extracted using both a commercial DNA extraction kit and a surfactant cracking DNA extraction method. Nested PCR and the RECCT-Cay method were used for detection. Nested-PCR amplification based on the 18S rRNA gene locus of *C. cayetanensis* was performed according to a previous study [28]. The primers used are listed in the Additional file 1: Supplementary Table S1. The PCR products were detected using 1.0% agarose gel electrophoresis with DNAGREEN (Tiandz, Beijing, China) at 100 V for 25 min. All positive PCR products were sent to Sangon Biotech (Zhengzhou, China) for bidirectional sequencing, aligning obtained sequences using MEGA 7.0 (https://www.megasoftware.net/), with reference sequences downloaded from GenBank.

### Development and evaluation of a portable suitcase based on the RECCT-Cay platform

To improve field applicability, a portable carrying case was developed and designed. This suitcase contains all the reagents and equipment needed for the entire process (from sample collection to the presentation of the test results). Using the positive fecal samples of *C. cayetanensis* stored in our laboratory, the feasibility of the portable suitcase developed based on the RECCT-Cay platform was evaluated. According to the preset procedure, *C. cayetanensis* was extracted from the samples using the surfactant cracking method, and the RECCT-Cay detection method was then employed.

## Results

### Rapid detection strategy of C. cayetanensis based on the RECCT-Cay

**Fig. 1:**
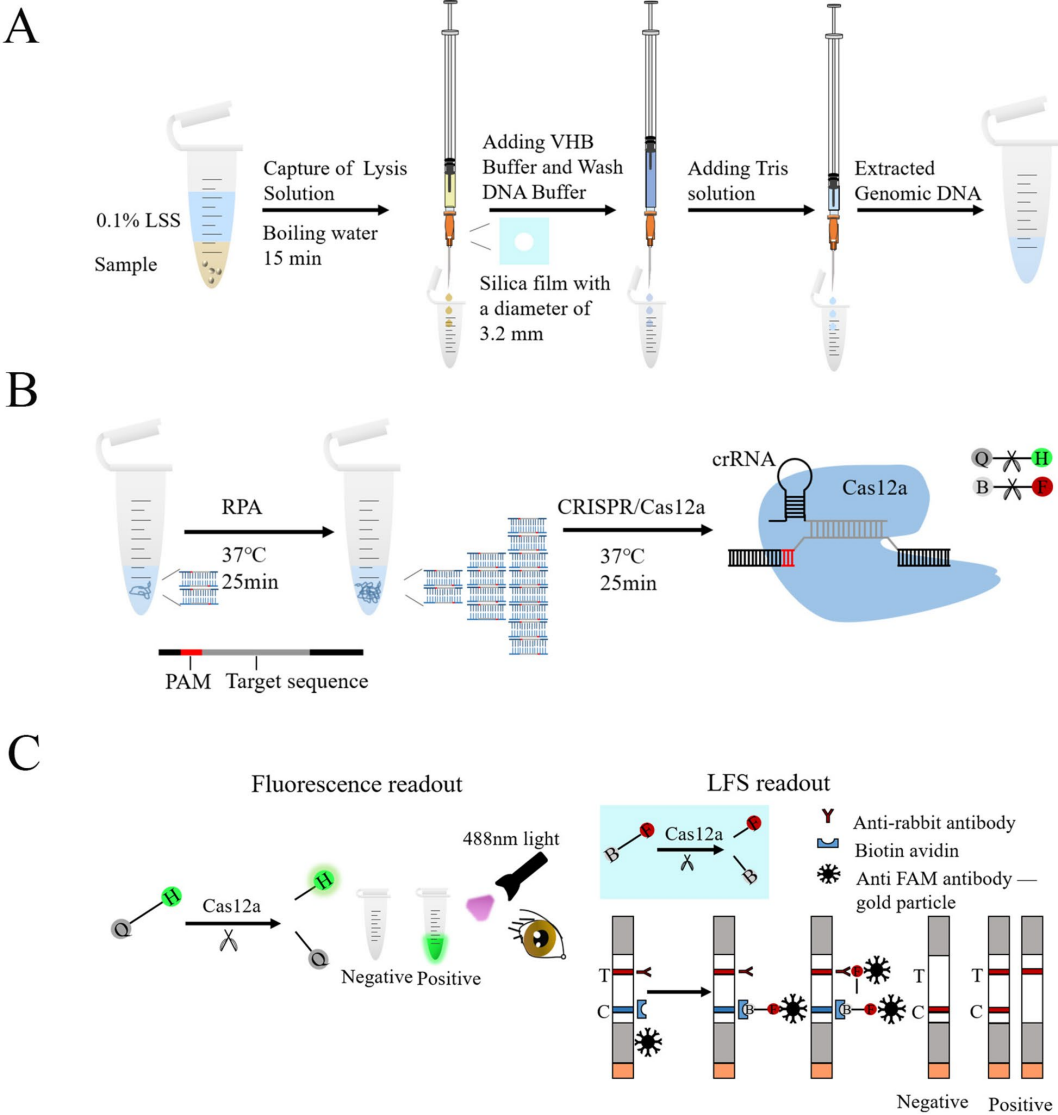
Schematic of the RECCT-Cay platform. **A** Rapid extraction of *C. cayetanensis* DNA from fecal samples by surfactant lysis. **B** Schematic of the RPA-CRISPR/Cas12-based diagnostic workflow. H-Q: HEX-BHQ, F-B: FAM-Biotin. **C** Readout of the RPA-CRISPR/Cas12a-based detection by a fluorescent reader or lateral flow strip (LFS)

To enable rapid and on-site diagnosis, we developed a comprehensive strategy that included genomic DNA extraction, RPA amplification, and detection of *C. cayetanensis* using CRISPR/Cas12-based fluorescence detection and lateral flow strip (LFS) readout methods. First, crude extraction of fecal genomic DNA was performed, followed by amplification of the target fragment via the RPA. Then, LbCas12a, crRNA, and the target dsDNA sequence with the TTV PAM sequence formed an LbCas12a-crRNA-dsDNA ternary complex. Recognition of the target dsDNA by the designed crRNA can induce activation of LbCas12a, which subsequently cleaves HEX-12N-BHQ1 to emit fluorescence at 520 nm under 488 nm light or cleaves the 6-FAM-12N-Biotin reporter to display visible T-lines on LFS (Fig. 1).

### Development of RPA primers and crRNA

To select the appropriate target sequence, we chose the *Cytb* and *Cox3* genes as target sites, as they are conserved in *C. cayetanensis* and specific relative to other parasites. Using the Prime-Blast online tool, we designed two pairs of RPA primers for each gene (Additional file 1: Supplementary Table S1), and the primers were synthesized by Sangon Biotech (Zhengzhou, China). After RPA amplification, analysis of the brightness and clarity of target bands in agarose gel electrophoresis revealed that RPA-F/R1b and RPA-F/R2a were the optimal primers, which were thus used in subsequent experiments. (Additional file 1: Supplementary Fig. S4).

**Fig. 2:**
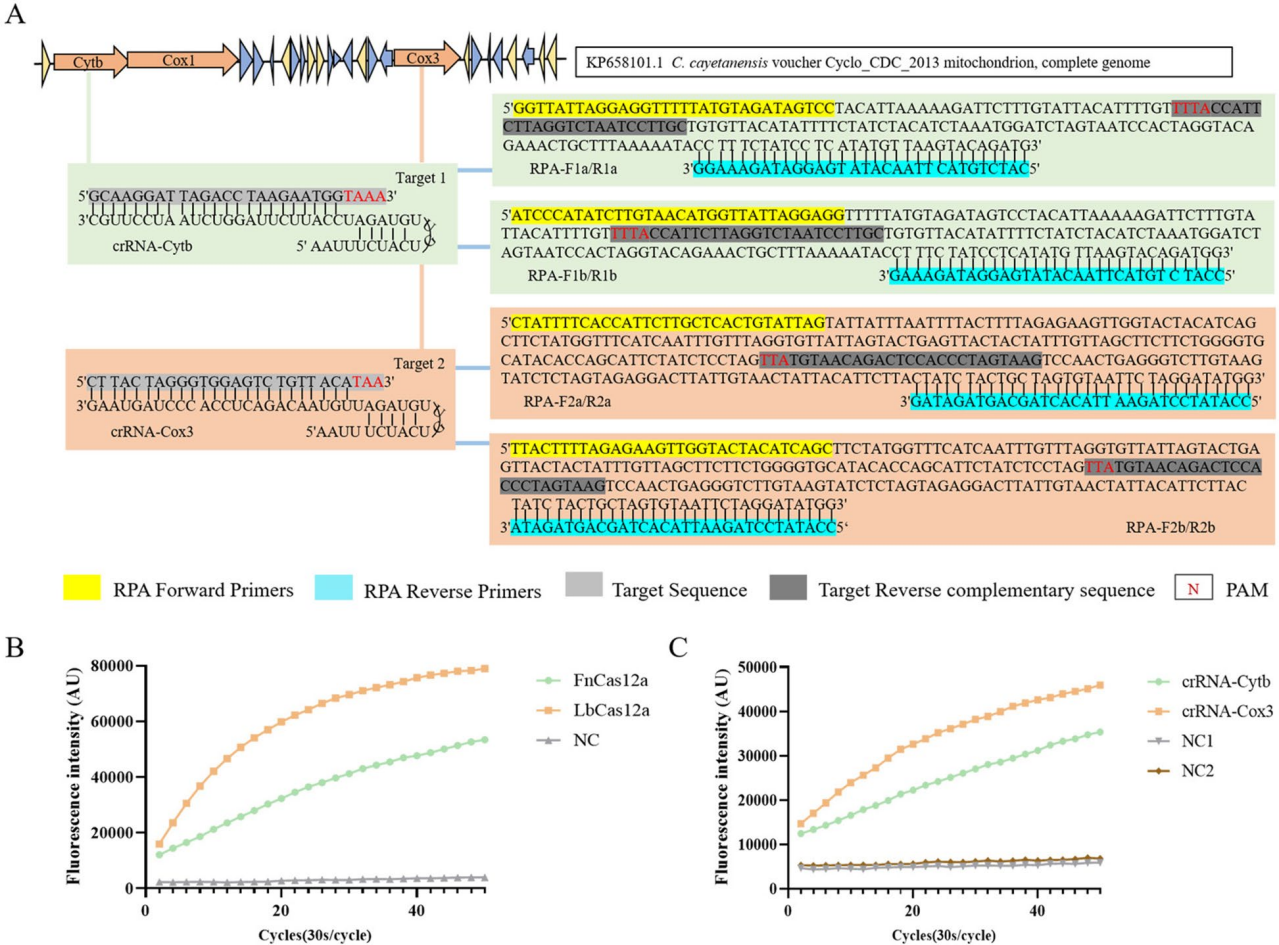
Screening of RPA amplification primers and crRNA. **A** The mitochondrial genome map shows the target site of the *C. cayetanensis* *Cytb* and *Cox*3, and it also shows the sequence of crRNA and the location of the RPA primer sequence in this assay. **B** Real-time fluorescence intensity curve obtained by combining crRNA-*Cox*3 with LbCas12a and FnCas12a, respectively. **C** Real-time fluorescence intensity curves obtained by combining LbCas12a combined with crRNA-*Cox*3 and crRNA-*Cytb*, respectively

The crRNAs were designed on the basis of a T-rich protospacer adjacent motif (PAM). The PAM sequence of FnCas12a is TTN, while that of LbCas12a is TTTN [29]. However, previous research has shown that LbCas12a can also recognize nonstandard PAM (such as CTTA, TCTA, and TTCA) [30]. Therefore, we selected a target sequence of 24 nucleotides located next to the TTA PAM design and synthesized a crRNA named crRNA-*Cox*3 (Fig. 2A, Additional file 1: Supplementary Fig. S5). By combining crRNA-*Cox*3 with LbCas12a and FnCas12a, we found that LbCas12a exhibited higher trans-cleave activity (Fig. 2B). Then, we selected a 23-nt target sequence located next to the TTTA PAM design and synthesized a crRNA-*Cytb* (Fig. 2A, Additional file 1: Supplementary Fig. S5). By combining crRNA-*Cox*3 and crRNA-*Cytb* with LbCas12a, we found that crRNA-*Cox*3 exhibited higher fluorescence values (Fig. 2C). Therefore, crRNA-*Cox*3 and LbCas12a were chosen to establish the system.

### Feasibility verification of the RECCT-Cay platform

**Fig. 3:**
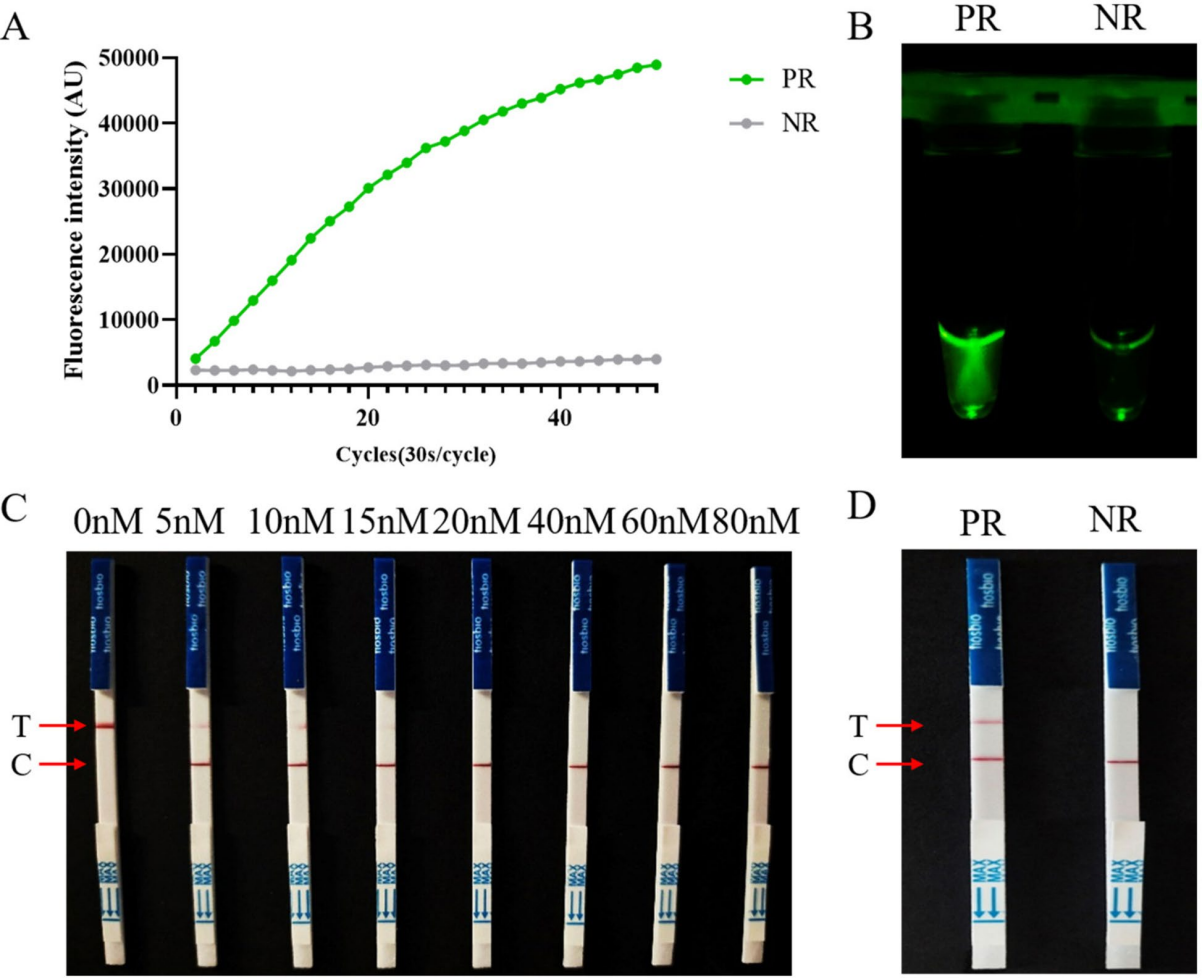
Establishment of a positive amplification system for *C. cayetanensis* detection. **A** Real-time fluorescence intensity curves of RPA-CRISPR/Cas12a-based detection involving H-Q reporter. **B** An obvious fluorescence signal of the positive sample was observed under UV light by the naked eye. PR, Positive results; NR, Negative results. **C** Optimization of the reporter concentration for RPA-CRISPR/Cas12a-based LFS detection. Various concentrations (0, 5, 10, 15, 20, 40, 60, 80 nM) of F-B reporter were tested. **D** An obvious T-line of the positive sample was observed by the naked eye. PR: Positive results, NR: Negative results

To preliminarily test the feasibility of the proposed RECCT-Cay platform, we used RPA primers, crRNA, and LbCas12a to perform a fluorescence detection assay. We recorded the fluorescence intensity every 30 s, and the real-time fluorescence curve is shown in Fig. 3A. The results indicated that 25 min was sufficient to observe a significant difference in fluorescence intensity between the positive and negative samples. Moreover, a clear, naked-eye-visible difference in fluorescence was observed between positive and negative samples under the blue light instrument (Fig. 3B).

To be able to diagnose *C. cayetanensis* in the field, we use LFS, which allow the results to be read without the need for instruments and skilled technicians. In the LFS detection method, to avoid false-positive results, we explored the optimal probe concentration. False-positive results were observed when reporter genes at 15 nM or lower concentrations were used, which were eliminated when reporter genes at 20 nM or higher were used (Fig. 3C). Therefore, 20 nM was selected as the optimal reporter concentration. We observed that positive samples had noticeable test lines (T-line) while negative samples only showed quality control lines (C-line) (Fig. 3D). The results demonstrated successful construction of a detection system for *C. cayetanensis*.

### Sensitivity and specificity of the RECCT-Cay platform

**Fig. 4:**
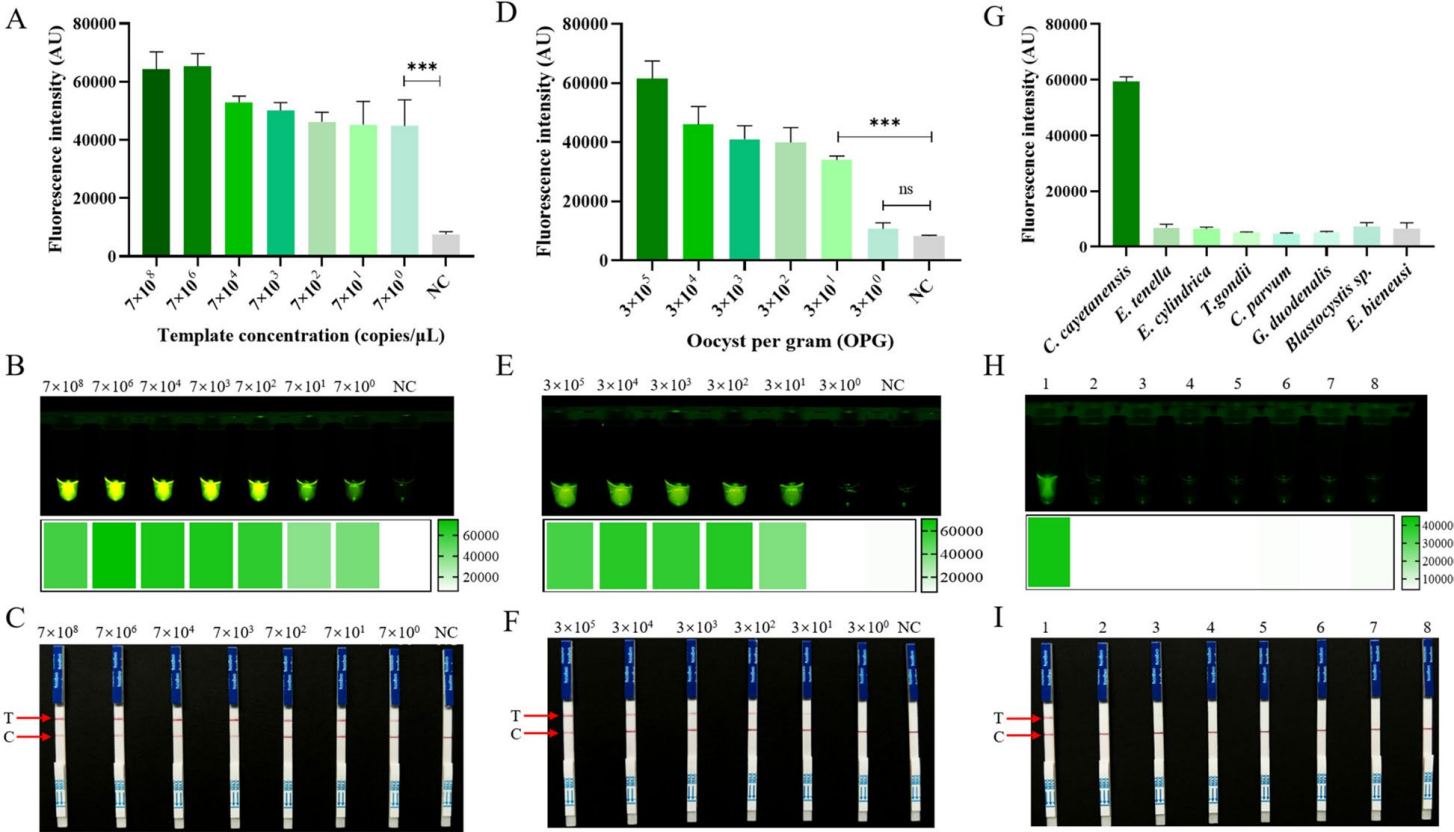
Sensitivity and specificity of the RECCT-Cay platform. The sensitivity and specificity of the RECCT-Cay platform. Nucleic acid sensitivity: **A** Fluorescence intensity values read by the qPCR instrument, **B** Fluorescence results under a blue light instrument, **C** LFS results; oocyst sensitivity: **D** fluorescence intensity values read by the qPCR instrument, **E** fluorescence results under a blue light instrument, **F** LFS results; specificity: **G** Fluorescence intensity values read by the qPCR instrument, **H** fluorescence results under a blue light instrument, **I** LFS results. 1–8: *C. cayetanensis*, *E. tenella*, *E. cylindrica*, *T. gondii*, *C. parvum*, *G. duodenalis*, *Blastocystis* sp., and *E. bieneusi*, respectively. Data in **A**, **D**, and **G** are presented as the mean ± standard deviation of three replicates. ***: *P* < 0.001; ****: *P* < 0.0001; *ns* not statistically significant

To determine the LOD of the established POCT system, pure parasitic DNA samples were diluted across a gradient of 7 × 10^9^–7 × 10^0^ copies/μL, and the POCT assay was then performed for each diluted sample. The samples with 7 copies/μL and above had a significant fluorescence signal compared to the negative control samples (Fig. 4A and B, Additional file 1: Supplementary Fig. S6). Similar results were observed in the LFS assay, with a LOD of 7 copies/μL (Fig. 4C). Subsequently, different concentrations of DNA (OPG of 3 × 10^5^–3 × 10^0^) were tested. The samples of 3 × 10^1^ or higher had a significant fluorescence signal (Fig. 4D and E, Additional file 1: Supplementary Fig. S7). Similar results were observed in the LFS assay based on RECCT-Cay, with samples with an OPG of 3 × 10^1^ or higher showing T-lines (Fig. 4F). The LOD of the constructed RECCT-Cay platform was determined to be 30 oocysts per gram of stool.

To test the specificity of the established POCT system, different parasites including *T. gondii, C. parvum, G. duodenalis, Blastocystis* sp., *E. bieneusi*, *E. tenella*, and *E. cylindrica* were recruited alongside *C. cayetanensis* as the positive group. In the fluorescence assay, only *C. cayetanensis* showed a visible fluorescence signal (Fig. 4G and H, Additional file 1: Supplementary Fig. S8). Similarly, in the LFS assay, only *C. cayetanensis* showed clear T-lines (Fig. 4I). The RECCT-Cay platform constructed in this study has high specificity, effectively detecting and distinguishing *C. cayetanensis* from other parasitic species.

### Performance of the RECCT-Cay platform in clinical samples

**Fig. 5:**
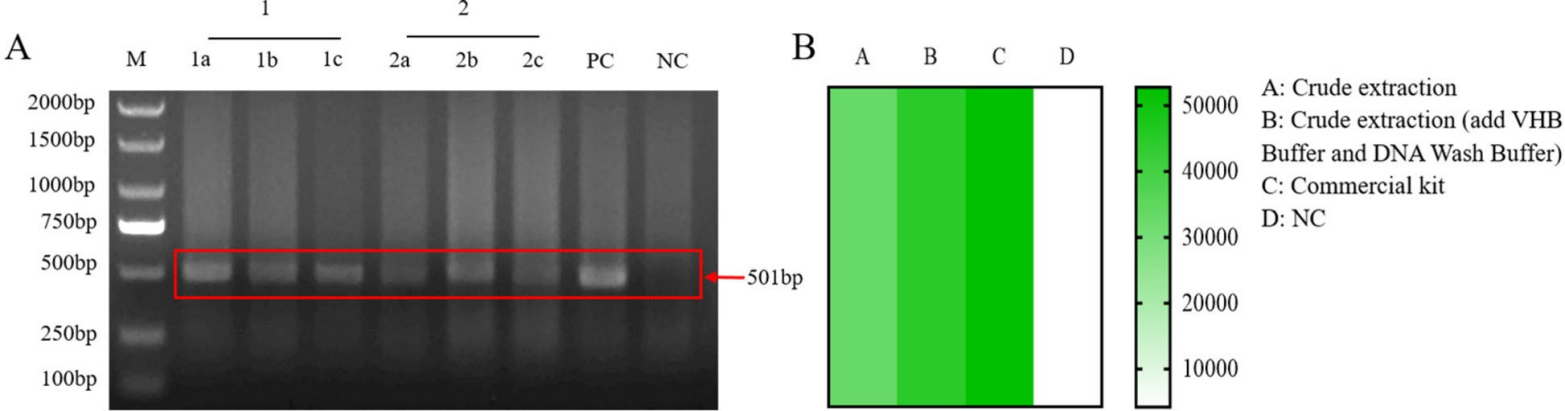
Comparison of the effects of different methods for extracting fecal genomic DNA. **A** Evaluation of the optimized surfactant pyrolysis method by agarose gel electrophoresis. The nested PCR reaction was performed with two groups: group 1, where VHB Buffer and DNA Wash Buffer were added to purify DNA from crude extraction; and group 2, where VHB Buffer and DNA Wash Buffer were not added to extract DNA. PC, Positive control; NC, Negative control. **B** Fluorescence detection based on RECCT-Cay was used to compare different genomic DNA extraction methods

To eliminate the need for large centrifuges and specialized equipment when extracting genomic DNA from clinical fecal samples, we compared the extraction effects of the surfactant cracking method and commercial kit extraction method for *C. cayetanensis*. The extraction efficiency was evaluated according to the brightness of the target band in gel electrophoresis (Fig. 5A) and the fluorescence signal (Fig. 5B). Adding VHB Buffer and DNA Wash Buffer enhanced the extraction efficiency of the surfactant cracking method. Although the efficiency of DNA extraction by surfactant cracking was slightly lower than that of commercial kit extraction (Fig. 5B), the latter is time-consuming, complex, and requires large equipment. Therefore, we finally selected the surfactant cracking method for genomic DNA extraction from clinical stool samples.

**Fig. 6:**
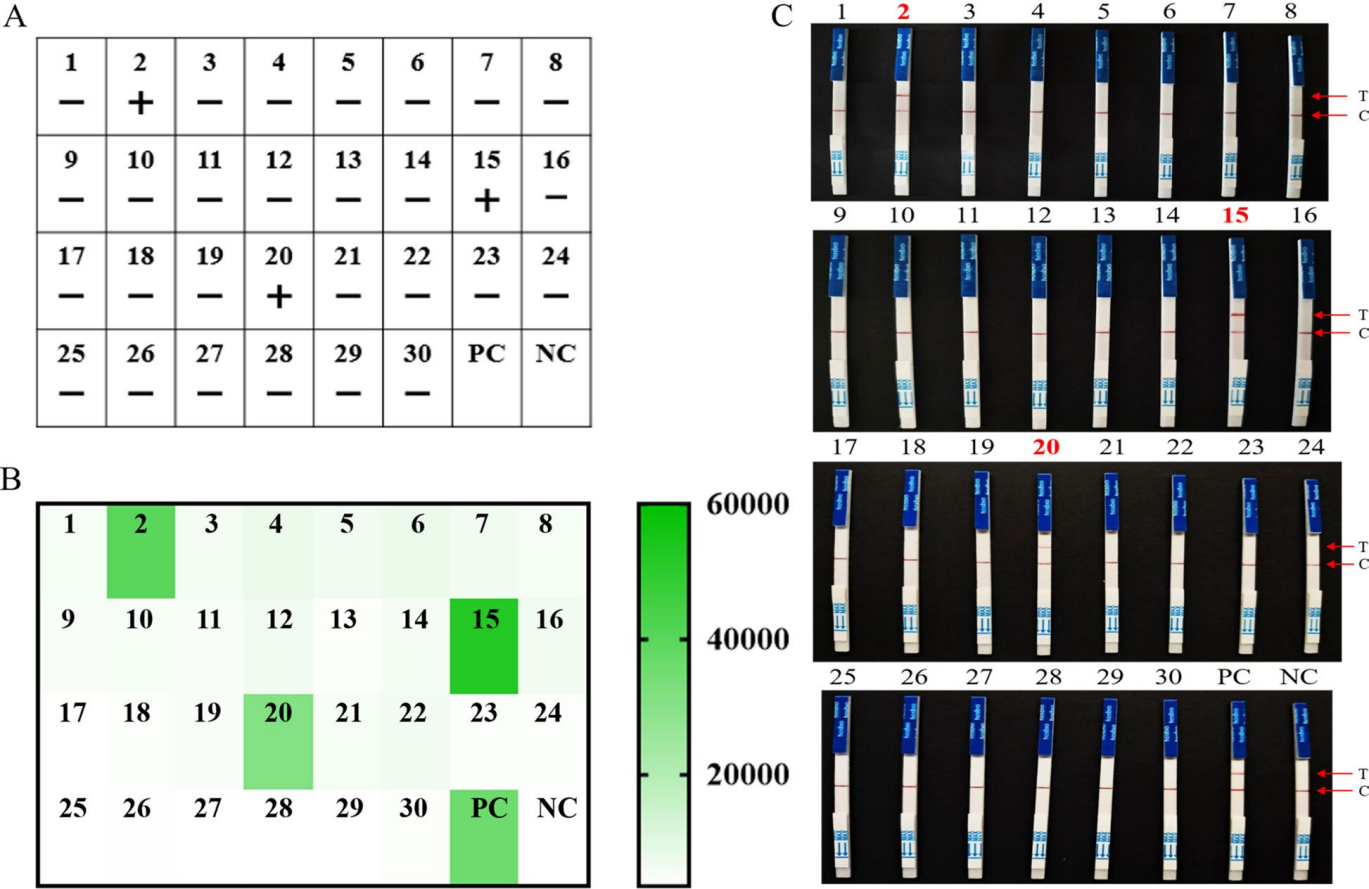
Validation of RECCT-Cay-based detection of *C. cayetanensis* in clinical human fecal samples. **A** 30 clinical fecal samples from humans were tested using the conventional nested PCR sequencing method. **B** The fluorescence detection and **C** LFS detection based on RECCT-Cay

Subsequently, to evaluate the clinical utility of the RECCT-Cay platform, fresh stool samples were collected from 30 hospitalized patients. All 30 samples were first tested by nested PCR, and three were positive for *C. cayetanensis* (Fig. 6A, Additional file 1: Supplementary Fig. S9). Both the fluorescence assay (Fig. 6B) and the LFS assay (Fig. 6C) yielded consistent results with the reference nested PCR, indicating that RECCT effectively detects *C. cayetanensis* from crude DNA extracts.

### Portable suitcase for rapid detection based on the RECCT-Cay platform

**Fig. 7:**
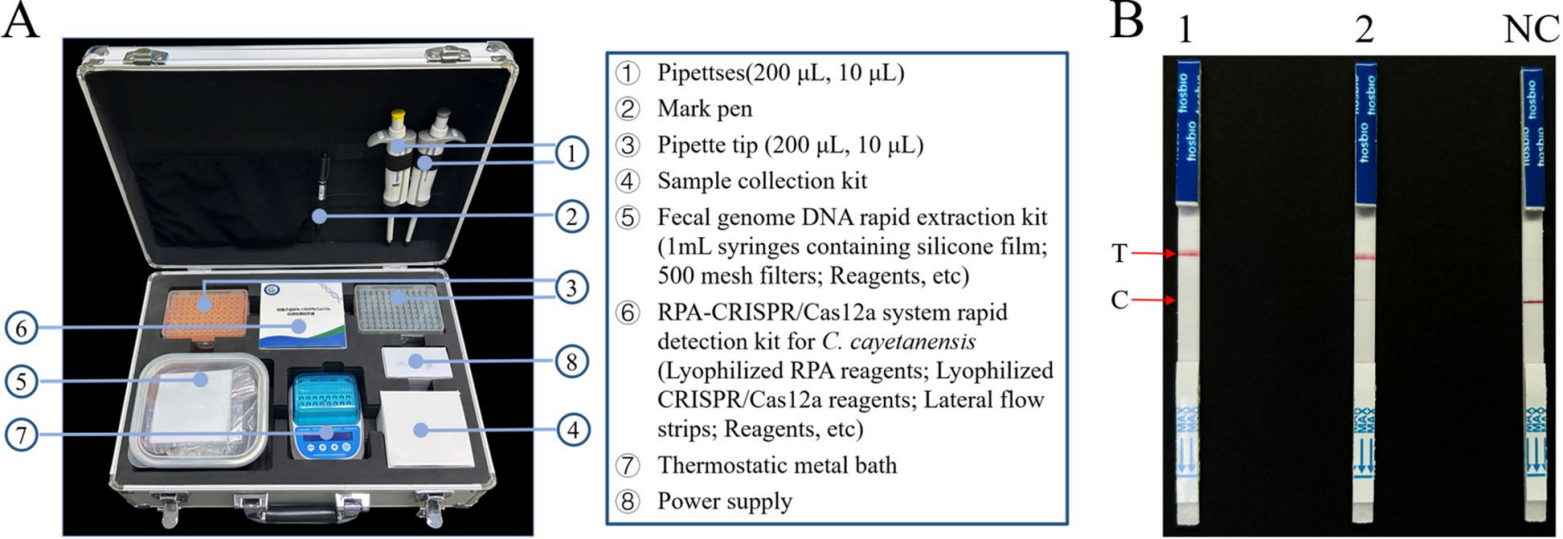
Development of a portable suitcase to detect *C. cayetanensis*. **A** Photos of the newly developed portable suitcases for point-of-care applications. **B** *C. cayetanensis* was determined by LFS assay detection. 1–2: *C. cayetanensis* PC, positive control; NC, negative control

To improve field applicability, we developed a portable suitcase on the basis of the RECCT-Cay platform. Measuring 44 × 32 × 17 cm (Fig. 7A), the portable suitcase includes: a sample collection kit, a fecal genome DNA rapid extraction kit, and the RPA-CRISPR/Cas12a detection kit (lyophilized RPA, lyophilized CRISPR/Cas12a reagents, and LFS). To facilitate storage and transportation, liquid RPA and CRISPR/Cas12a reagents were prepared in lyophilized powder form (Additional file 1: Supplementary Fig. S10). The feasibility of the portable suitcase was evaluated using preserved *C. cayetanensis*-positive fecal samples from our laboratory. For the LFS test, the positive sample exhibited a clear T-line, while the negative sample only showed a C-line (Fig. 7B). For the fluorescence detection method of the RECCT-Cay platform, a clear difference between the positive and negative samples was visible to the naked eye under a blue light meter (Additional file 1: Supplementary Fig. S11A). The results of our present method are consistent with those obtained by nested PCR, which confirms its efficacy for on-site diagnosis (Additional file 1: Supplementary Fig. S11B).

## Discussion

*Cyclospora cayetanensis* is an emerging food- and water-borne pathogen that causes endemic and epidemic human diarrhea worldwide [31]. *C. cayetanensis* has caused several foodborne outbreaks in many countries, including the USA, Mexico [32, 33], and Canada [34]. The newly developed method with high sensitivity and specificity can facilitate the detection of oocysts in clinical diagnosis and food. The researchers found that *C. cayetanensis* was more likely to occur in low-income countries and children [10]. Microscopy and PCR (real-time qPCR or conventional PCR) are commonly used for the laboratory diagnosis of *C. cayetanensis* [35]. Although PCR-based methods are highly accurate and suitable for high-throughput screening, they are limited by the need for sophisticated equipment, specialized personnel, and time-consuming procedures. These shortcomings hinder its practical application, especially in resource-limited environments. Therefore, there is a growing demand for more efficient, portable, and cost-effective diagnostic solutions. Our study addresses this urgent need by establishing a rapid, portable RECCT-Cay platform, providing a simple and effective alternative for practical field use.

Rapid portable pathogen detection methods based on the CRISPR/Cas system have been developed for *Leptospira* [36], coronavirus disease 19 (COVID-19) [37], *Burkholderia pseudomallei* [38], *C. parvum* [25], and *T. gondii* [39], and have satisfactory diagnostic performance. In this study, we combined RPA amplification and the CRISPR/Cas12a system to establish a stable, rapid, and sensitive method for *C. cayetanensis* detection. First, the target sequence is amplified by RPA, then Cas12a trans-cleavage activity is utilized, and finally, fluorescence readout and an LFS biosensor are used for signal detection. The results showed that the constructed RECCT-Cay platform exhibited high sensitivity and specificity (Fig. 4). This method represents a novel application for detecting *C. cayetanensis*, and is developed to complete the entire process within just 1 h at 37 °C, potentially using body temperature. Without the need for specialized equipment, the final detected signal can be clearly interpreted by the naked eye.

It is worth noting that we carefully screened the optimal target sites and designed the best RPA primers and crRNA. The commonly used genetic loci of *C. cayetanensis* include 18S rRNA, ITS, or HSP70 [2, 40–42]. The 18S rRNA gene sequence cannot distinguish *C. cayetanensis* from *Eimeria* spp., representing a major challenge in water and environmental detection [35]. ITS gene sequences are highly variable among different samples [43], and no conserved target sequences could be screened. The HSP70 gene locus is a low-copy gene, which reduces detection sensitivity [44]. Finally, we screened the best RPA primers and crRNA at the *Cox3* gene locus in the mitochondrial genome (approximately 6200 bp). The results show that the method constructed in this study has high specificity, enabling differentiation from *Eimeria* spp. (which are genetically closely related), laying a solid foundation for future detection of *C. cayetanensis* in food and water. In addition, it exhibits strong sensitivity, with a LOD of 7 copies/μL or 30 oocysts per gram of stool. Although the detection limit of nested PCR amplification by Resendiz-Nava et al. was as low as 1 *C. cayetanensis* OPG per sample, it showed cross-reactivity with *Eimeria* species [45]. The FDA has developed a mit3PCR for the detection of *C. cayetanensis*. When a minimum of 20 oocysts are inoculated, the positive detection rate can reach 100%, which is comparable to the sensitivity of this study [46]. Richins T et al. developed a single-tube nested qPCR for the detection of *C. cayetanensis*, with a LOD as low as 0.613 oocysts/g, but cross-reacted with *Eimeria* spp [47]. Costa ADT developed a ready-to-use qPCR for the detection of *C. cayetanensis*, with a LoD_95_% of 17.54 copies/reaction. The positive detection rate was 100% in the 200 oocysts /μL, while a few false negative results occurred in the 5–10 oocysts /μL. This method emphasizes the need for frozen transportation, yet the reagents still need to be stored at 2–8 °C [48].

In this study, fluorescence sensing and LFS biosensor were employed for signal detection, addressing the demand for visual detection methods—particularly critical for molecular diagnosis in remote areas where instrumentation may be unavailable [49]. When the HEX-12N-BHQ1 reporter was used, the results could be visualized with the naked eye under blue light. When the 6-FAM-12N-Biotin reporter was used, results were visually detectable via the LFS biosensor. Visual inspection enables simple and rapid interpretation of results without requiring professional technicians, large-scale equipment, or power supplies, rendering the RECCT-Cay platform a practical diagnostic tool. Importantly, we integrated a rapid fecal DNA extraction method (the 0.1% LSS lysis method, completed within 20 min) with the RECCT assay. To validate the clinical applicability of the RECCT-Cay platform, we extracted crude DNA from 30 human fecal samples. Three positive samples were identified by both fluorescence and LFS assays, with results showing high consistency with those of nested PCR targeting the 18S rRNA locus. These findings confirm the reliability of the RECCT-Cay platform established in this study. As a detection method for *C. cayetanensis*, the approach developed herein exhibits improvements in simplicity, sensitivity, specificity, efficiency, and equipment independence.

Finally, we developed and designed a portable suitcase integrated with the required equipment and lyophilized reagents, which meets the needs of field testing. This portable suitcase, designed for *C. cayetanensis* detection, offers a “one-stop service” covering sample collection, rapid fecal DNA extraction, and the RECCT-Cay assay. It can be applied in remote areas or field settings for clinical testing. Furthermore, the device is compact and portable, requiring no large-scale equipment—an important advancement toward fast, affordable, and accessible POCT in the future.

However, this method has certain limitations. First, although the isothermal amplification feature of RPA reduces equipment costs, the cost of RPA reagents remains relatively high. Future efforts should focus on reducing costs through mass production or the use of single-tube systems capable of detecting multiple pathogens. Second, we adopted a two-step approach to separate RPA from CRISPR/Cas12a reactions. However, the need to open the reaction tube after RPA amplification increases the risk of aerosol contamination. This two-step process could be optimized into a single-pot reaction to mitigate such contamination. Third, extracting parasitic genomic DNA from fecal samples is more challenging compared to extracting viral or bacterial DNA. The efficiency of rapid fecal genomic DNA extraction using the LSS lysis method thus requires further improvement in future experiments. Despite these limitations, the RPA-CRISPR/Cas12a test kit developed for *C. cayetanensis* exhibits high sensitivity and robustness.

## Conclusions

This study is the first to employ the RPA isothermal amplification combined with CRISPR/Cas12a technology for the detection of *C. cayetanensis*. The method exhibits excellent specificity, showing no cross-reactivity with closely related species such as *Eimeria* spp. It also demonstrates high sensitivity, with a LOD of 7 copies/μL or 30 oocysts per gram in stool samples. In addition, we have designed and developed a portable suitcase integrating essential equipment and lyophilized reagents, eliminating the need for large-scale instruments and laboratory settings. This suitcase enables applications in remote areas or on-site clinical testing, offering the advantage of a “one-stop service.” The proposed RECCT-Cay platform is rapid, convenient, and efficient, and can be widely applied to various sample types, including food, soil, water, and feces. Overall, this approach lays an important foundation for the future development of rapid and cost-effective POCT.

## Supplementary Information

Additional file 1: Figure S1. Homemade DNA banding column. Figure S2. Gel electrophoresis of annealing, transcription and purified products. Figure S3. Result of PCR amplification based on the Cox3 locus of C. cayetanensis. Figure S4. Evaluation of RPA primers by agarose gel electrophoresis. Figure S5. Select Target sequence. Figure S6. The real-time fluorescence intensity curve of the nucleic acid sensitivity test based on the fluorescence detection of RECCT-Cay. Figure S7. The real-time fluorescence intensity curve of the oocyst sensitivity test based on the fluorescence detection of RECCT-Cay. Figure S8. The real-time fluorescence intensity curve of the specificity test of the RPA-CRISPR/Cas12a-based fluorescence detection. Figure S9. Results of C. cayetanensis amplification in human fecal samples based on 18S rRNA gene locus. Figure S10. Images of RPA and CRISPR/Cas12a lyophilized reagents in an eight-row tube tubes. Figure S11. Evaluating the feasibility of a portable suitcase test using a positive stool sample for C. cayetanensis. Table S1. Nucleotide sequences used in this study. Table S2. Information of non-target parasite DNA samples. Table S3. Information of 30 fecal clinical samples from inpatients. Table S4. Concentration determination of purified crRNA.

## Data Availability

Data supporting the main conclusions of this study are included in the manuscript.

## Abbreviations

CRISPR: Clustered regularly interspaced short palindromic repeats
Cas: CRISPR-associated
RPA: Recombinase polymerase amplification
RECCT-Cay: Recombinase polymerase amplification and the CRISPR/Cas12a system detection for *C. cayetanensis*
POCT: Point-of-care testing
LFS: Lateral flow strip
OPG: Oocysts per gram
PCR: Polymerase chain reaction
DETECTR: DNA endonuclease-targeted CRISPR trans reporter
crRNA: CRISPR RNA
dsDNA: Double-strand DNA
ssDNA: Single-stranded DNA
PAM: Protospacer-adjacent motif
nt: Nucleotide
HEX: Hexachloro fluorescein
FAM: Fluorescein amidite
BHQ1: Black hole quencher 1
LSS: N-Lauroylsarcosine sodium salt
PBS: Phosphate buffer solution
*Cytb*: Cytochrome b
*Cox*3: Cytochrome c oxidase subunit 3
LOD: Limit of detection
FDA: Food and drug administration
