# Supplementary material for: Development of a field-deployable RPA-CRISPR/Cas12a assay for the detection of Cyclospora cayetanensis in human feces
Source: Parasit Vectors. 2025 Nov 28;19:4. doi: 10.1186/s13071-025-07150-x (PMC12763960; doi:10.1186/s13071-025-07150-x)
Supplement: Supplementary file 1 — Additional file 1: Figure S1. Homemade DNA banding column. Figure S2. Gel electrophoresis of annealing, transcription and purified products. Figure S3. Result of PCR amplification based on the Cox3 locus of C. cayetanensis. Figure S4. Evaluation of RPA primers by agarose gel electrophoresis. Figure S5. Select Target sequence. Figure S6. The real-time fluorescence intensity curve of the nucleic acid sensitivity test based on the fluorescence detection of RECCT-Cay. Figure S7. The real-time fluorescence intensity curve of the oocyst sensitivity test based on the fluorescence detection of RECCT-Cay. Figure S8. The real-time fluorescence intensity curve of the specificity test of the RPA-CRISPR/Cas12a-based fluorescence detection. Figure S9. Results of C. cayetanensis amplification in human fecal samples based on 18S rRNA gene locus. Figure S10. Images of RPA and CRISPR/Cas12a lyophilized reagents in an eight-row tube tubes. Figure S11. Evaluating the feasibility of a portable suitcase test using a positive stool sample for C. cayetanensis. Table S1. Nucleotide sequences used in this study. Table S2. Information of non-target parasite DNA samples. Table S3. Information of 30 fecal clinical samples from inpatients. Table S4. Concentration determination of purified crRNA. [file 13071_2025_7150_MOESM1_ESM.docx]

**Additional file 1**

**Development of a Field-Deployable RPA-CRISPR/Cas12a Assay for the Detection of *Cyclospora cayetanensis* in Human Feces**

**Ziyang Qin^1,2,3^, Yilin Wang^1,2,3^, Mengqing Sun^1,2,3^, Qinglin Wang^1,2,3^, Junxia Duan^1,2,3^, Chunhao Gu^1,2,3^, Xinfu Zhang^1,2,3,4^, Fuchang Yu^5^, Yayun Wu^1,2,3^, Huiyan Xu^1,2,3^, Junqiang Li^1,2,3*^, Longxian Zhang^1,2,3*^**

1 College of Veterinary Medicine, Henan Agricultural University, Zhengzhou 450046, P. R. China

2 International Joint Research Laboratory for Zoonotic Diseases of Henan, Zhengzhou 450046, Henan Province, China

3 Key Laboratory of Quality and Safety Control of Poultry Products, Ministry of Agriculture and Rural Affairs, P.R. China

4 Gene Editing Center for Veterinary Medicine, Henan Agricultural University, Zhengzhou 450046, China

5 College of Animal Science and Technology, Tarim University, Alar, Xinjiang 843300, PR China

*Corresponding author: Longxian Zhang, College of Veterinary Medicine, Henan Agricultural University, No. 218 of Ping An Avenue, Zhengdong Newly-developed Area, Zhengzhou 450046, China. Tel: 86-371-56990163; Fax: 86-371-56990163; E-mail: zhanglx8999@henau.edu.cn

Junqiang Li, College of Veterinary Medicine, Henan Agricultural University, No. 218 of Ping An Avenue, Zhengdong Newly-developed Area, Zhengzhou 450046, China. Tel.: 86-371-56990363, Fax: 86-371-56990363, E-mail: lijunqiangcool@126.com

**
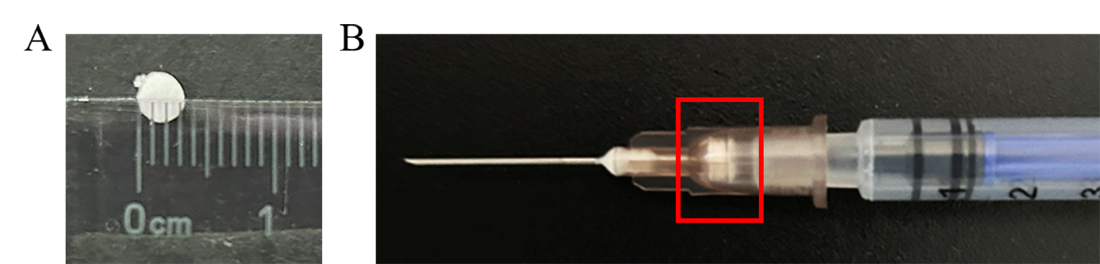
Fig. S1 Homemade DNA banding column.** A: The diameter of the punched silica membrane is about 3.2 mm. B: The homemade DNA banding column is assembled from a syringe needle and two pieces of punched silica membrane inside (in the red rectangle).


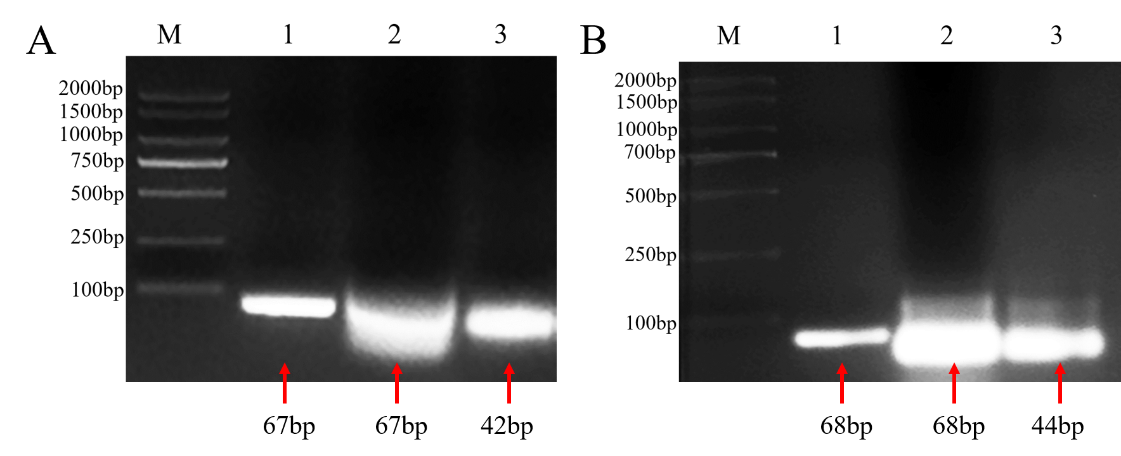


**Fig. S2 Gel electrophoresis of annealing, transcription and purified products.** (A: crRNA-*Cytb*; B: crRNA-*Cox*3). M: DL2000 DNA Marker; 1: Annealing product; 2: Transcription product; 3: Purified product.


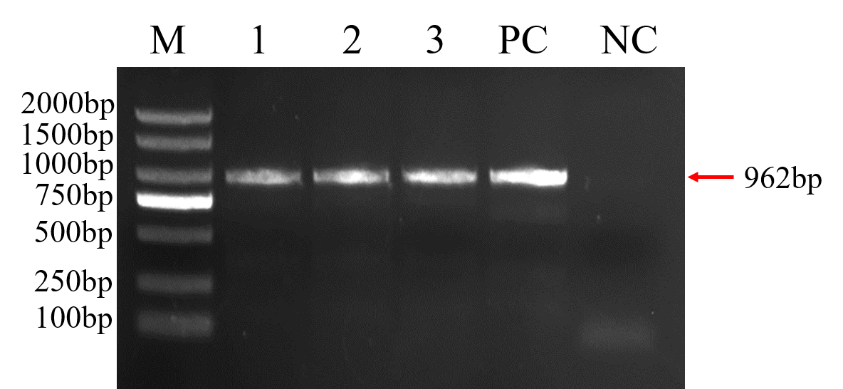


**Fig. S3 Result of PCR amplification based on the *Cox*3 locus of *C. cayetanensis*.** M: DL2000 DNA Marker; 1-3: Samples; PC: Positive control; NC: Negative control.


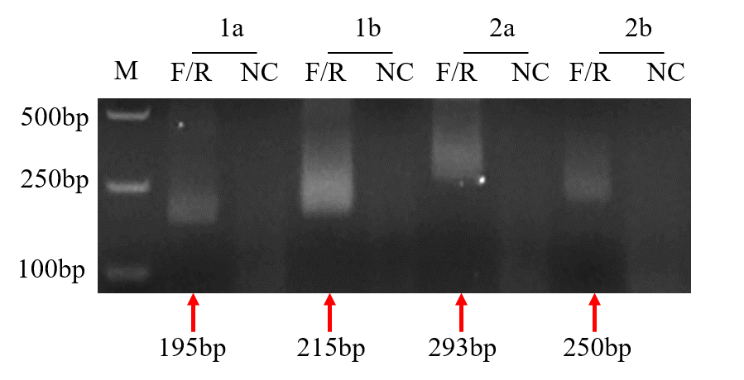
**Fig. S4 Evaluation of RPA primers by agarose gel electrophoresis.** RPA reaction is performed with 4 sets of primers. 1: RPA-F1a and RPA-R1a; 2: RPA-F1b and RPA-R1b; 3: RPA-F2a and RPA-R2a; 4: RPA-F2b and RPA-R2b; NC: Negative control.


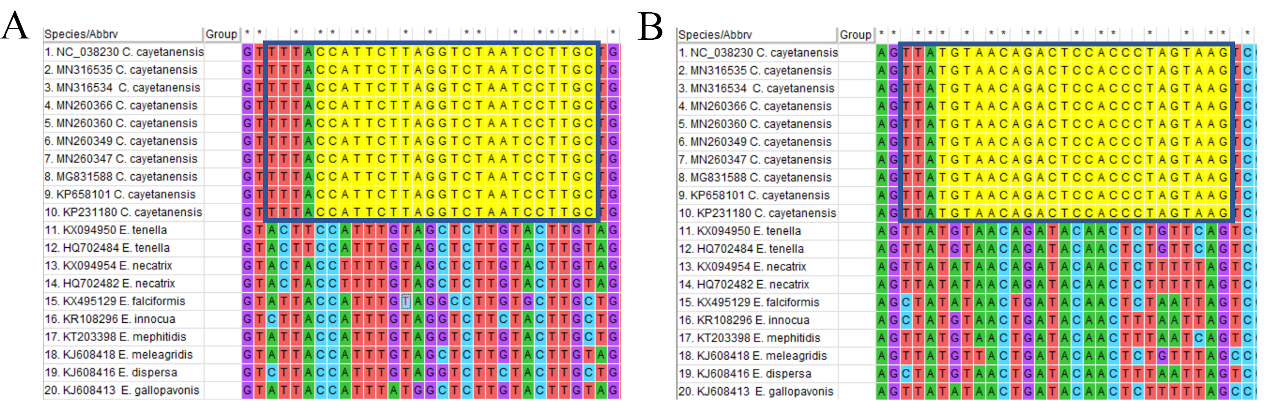


**Fig. S5 Select Target sequence.** A: The alignment of target sequence based on mitochondrial genome *Cytb* gene locus. B: The alignment of target sequence based on mitochondrial genome *Cox*3 gene locus.

**
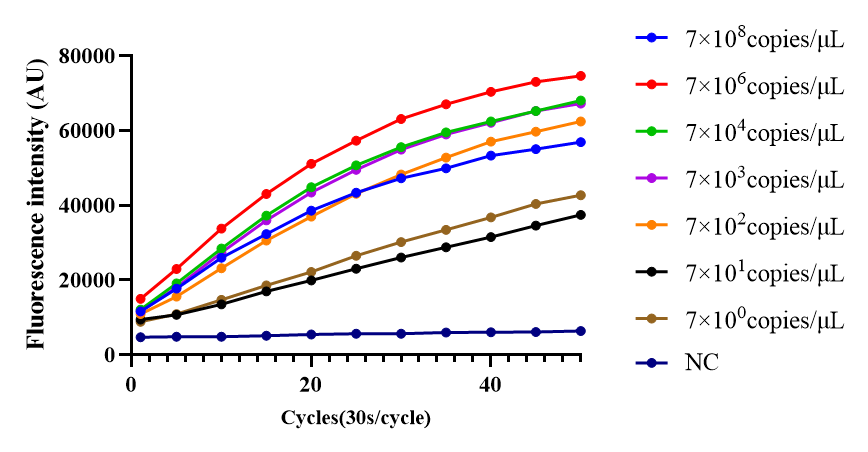
**

**Fig.S6 The real-time fluorescence intensity curve of the nucleic acid sensitivity test based on the fluorescence detection of RECCT-Cay.** NC: Negative control.

**
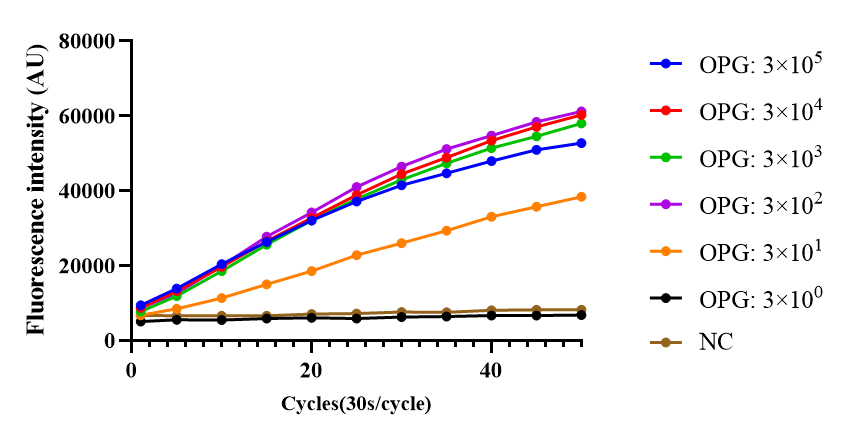
**

**Fig. S7 The real-time fluorescence intensity curve of the oocyst sensitivity test based on the fluorescence detection of RECCT-Cay.** NC: Negative control.

**
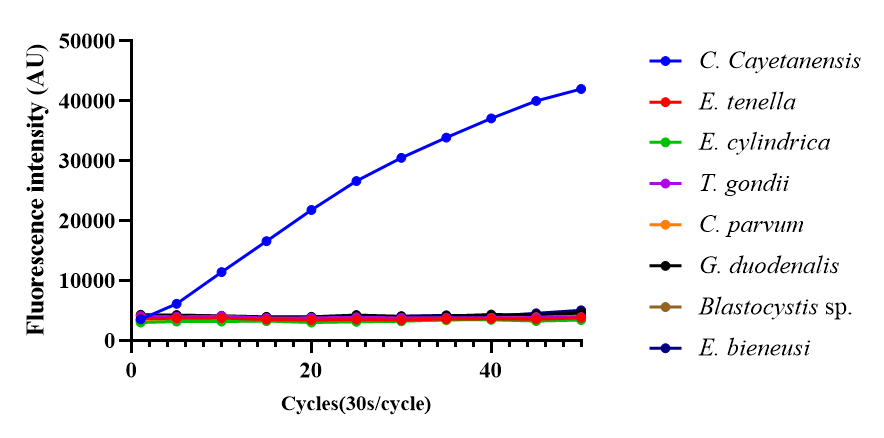
**

**Fig. S8 The real-time fluorescence intensity curve of the specificity test of the RPA-CRISPR/Cas12a-based fluorescence detection.**

**
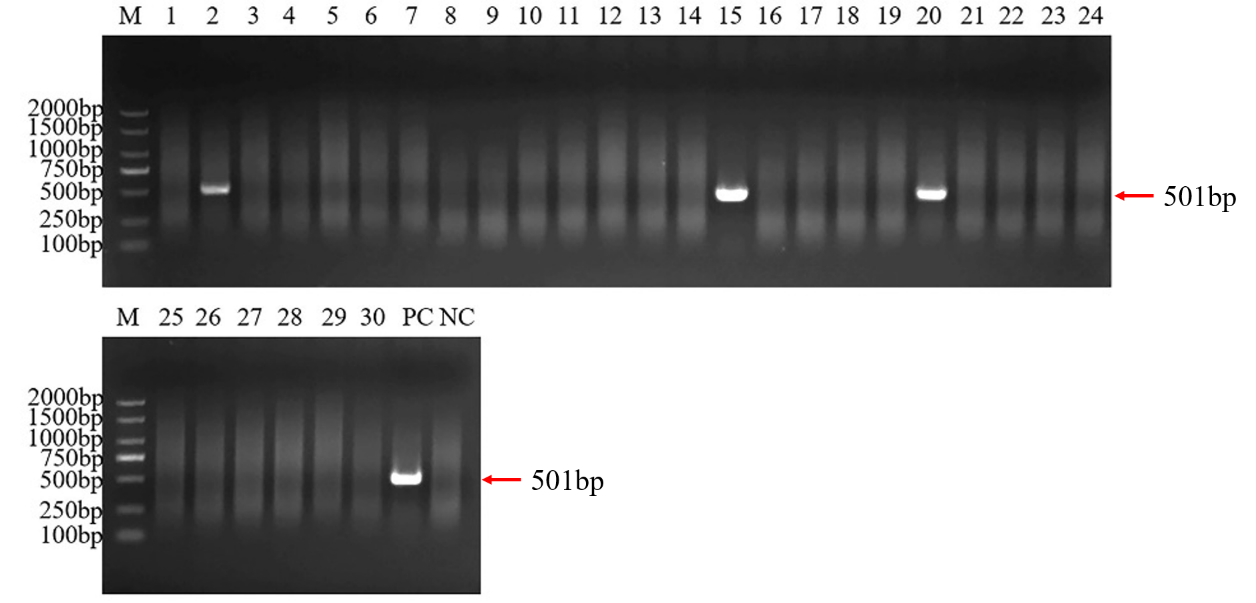
**

**Fig. S9 Results of *C. cayetanensis* amplification in human fecal samples based on 18S rRNA gene locus.** M: DL2000 DNA Marker; 1-30: Human fecal samples; PC: Positive control; NC: Negative control.

**
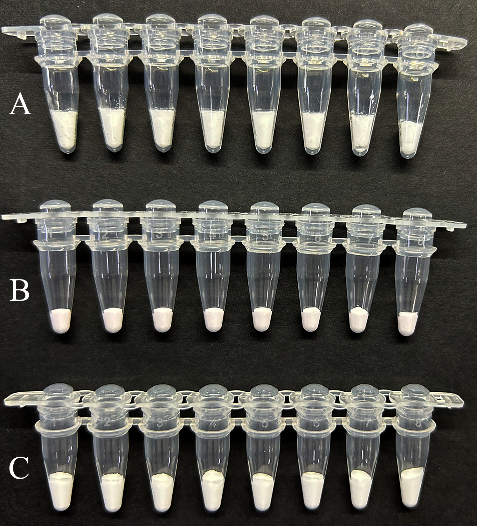
Fig. S10 Images of RPA and CRISPR/Cas12a lyophilized reagents in an eight-row tube tubes.** A: Lyophilized RPA reagents; B: Lyophilized CRISPR/Cas12a fluorescence detection reagent; C Lyophilized CRISPR/Cas12a LFS detection reagent.


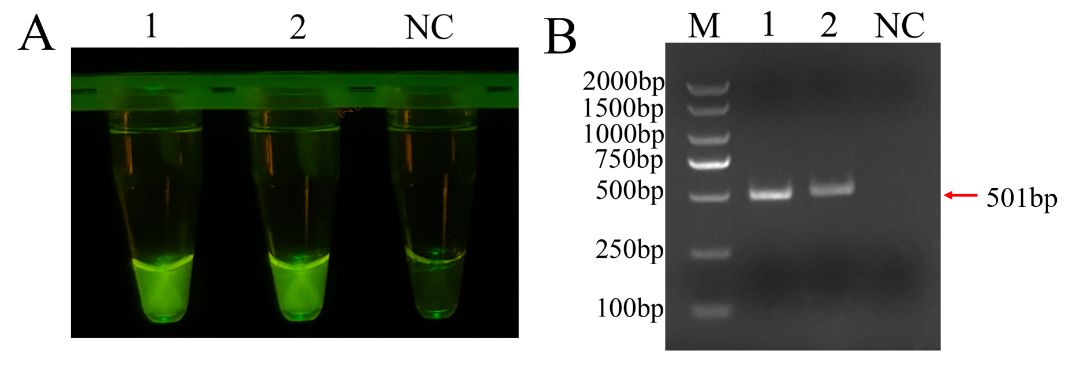


**Fig. S11 Evaluating the feasibility of a portable suitcase test using a positive stool sample for *C. cayetanensis*.** A: Fluorescence detection based on RPA-CRISPR/Cas12; B: Nested PCR detection based on the 18S rRNA gene locus; 1-2：Positive control; NC: Negative control.

**Table S1** **Nucleotide sequences used in this study.**

| Name | | Sequence (5’-3’) |
| --- | --- | --- |
| RPA primers | RPA-F1a | GGTTATTAGGAGGTTTTTATGTAGATAGTCC |
|  | RPA-R1a | CATCTGTACTTAACATATGAGGATAGAAAGG |
|  | RPA-F1b | ATCCCATATCTTGTAACATGGTTATTAGGAGG |
|  | RPA-R1b | CCATCTGTACTTAACATATGAGGATAGAAAG |
|  | RPA-F2a* | CTATTTTCACCATTCTTGCTCACTGTATTAG |
|  | RPA-R2a* | CCATATCCTAGAATTACACTAGCAGTAGATAG |
|  | RPA-F2b | TTACTTTTAGAGAAGTTGGTACTACATCAGC |
|  | RPA-R2b | CCATATCCTAGAATTACACTAGCAGTAGATA |
| ss crDNA | ss crDNA-F-*Cytb* | gaaatTAATACGACTCACTATAgggAATTTCTACTGTTGTA  GATCCATTCTTAGGTCTAATCCTTGC |
|  | ss crDNA-R-*Cytb* | GCAAGGATTAGACCTAAGAATGGATCTACAACAGTAG  AAATTcccTATAGTGAGTCGTATTAatttc |
|  | ss crDNA-F-*Cox*3 | gaaatTAATACGACTCACTATAgggAATTTCTACTGTTGTAGATTGTAACAGACTCCACCCTAGTAAG |
|  | ss crDNA-R-*Cox*3 | CTTACTAGGGTGGAGTCTGTTACAATCTACAACAGTAGAAATTcccTATAGTGAGTCGTATTAatttc |
| crRNA | crRNA-*Cytb* | AAUUUCUACUGUUGUAGAUccauucuuaggucuaauccuugc |
|  | crRNA-*Cox*3* | AAUUUCUACUGUUGUAGAUuguaacagacuccacccuaguaag |
| PCR primers | 18S rRNA-F1 | AATGTAAAACCCTTCCAGAGTAAC |
|  | 18S rRNA-R1 | GCAATAATCTATCCCCATCACG |
|  | 18S rRNA-F2 | AATTCCAGCTCCAATAGTGTAT |
|  | 18S rRNA-R2 | CAGGAGAAGCCAAGGTAGGCRTTT |
|  | *Cox*3-F | AGAAAACCTAAAATCATCATGT |
|  | *Cox*3-R | AAGTGAGTTCGCATGTTTAC |
| FAM-12N-Biotin reporter | | 6-FAM-NNNNNNNNNNNN-Biotin |
| HEX-12N-BHQ1 reporter | | HEX-NNNNNNNNNNNN-BHQ1 |

* Corresponds to the optimum crRNA and RPA primers used in this study.

**Table S2 Information of non-target parasite DNA samples**

| Parasite | Host | Sample source | Strain designation |
| --- | --- | --- | --- |
| *E. tenella* | Gallus gallus | A Poultry Farm in Kaifeng, Henan Province, China | HNKF2022-0057 |
| *E. cylindrical* | Cattle | A dairy farm in Xinxiang, Henan Province, China | HNXX2022-0342 |
| *T. gondii* | Hu sheep | A sheep farm in Huzhou, Zhejiang Province, China | TgZJ003 |
| *C. parvum* | Cattle | A dairy farm in Baoding, Hebei Province, China | C2HB-2023 |
| *Blastocystis* sp. | Human | A hospital in Zhengzhou, Henan Province, China | HNZZ2019-0883 |
| *G. duodenalis* | Human | A hospital in Kafr El Sheikh Province, Egypt | EgyGb045 |
| *E. bieneusi* | Human | A hospital in Zhengzhou, Henan Province, China | HNZZ2023-0002 |

**Table S3 Information of 30 fecal clinical samples from inpatients**

| Sample Number | Sample ID | Gender | Age | Department | Diarrhea Status |
| --- | --- | --- | --- | --- | --- |
| 1 | HNKF2023-0707 | Female | 19 | Gastroenterology Department | With Diarrhea |
| 2 | HNKF2023-1540 | Female | 38 | Gastroenterology Department | With Diarrhea |
| 3 | HNKF2023-1541 | Male | 65 | Oncology Department | With Diarrhea |
| 4 | HNKF2023-1554 | Male | 57 | Gastroenterology Department | With Diarrhea |
| 5 | HNKF2023-1575 | Male | 68 | General Surgery Department | Without Diarrhea |
| 6 | HNKF2023-1615 | Female | 55 | Cardiology Department | Without Diarrhea |
| 7 | HNKF2023-1633 | Male | 22 | Emergency Department | Without Diarrhea |
| 8 | HNKF2023-1672 | Male | 22 | Gastroenterology Department | With Diarrhea |
| 9 | HNKF2023-1688 | Male | 26 | Gastroenterology Department | With Diarrhea |
| 10 | HNKF2023-1689 | Male | 15 | Gastroenterology Department | With Diarrhea |
| 11 | HNKF2023-1707 | Male | 41 | Gastroenterology Department | Without Diarrhea |
| 12 | HNKF2023-1714 | Female | 41 | Gastroenterology Department | With Diarrhea |
| 13 | HNKF2023-1733 | Male | 22 | Gastroenterology Department | Without Diarrhea |
| 14 | HNKF2023-1758 | Female | 44 | Oncology Department | Not Recorded |
| 15 | HNKF2023-1777 | Male | 57 | Cardiology Department | Not Recorded |
| 16 | HNKF2023-1783 | Female | 54 | Cardiology Department | With Diarrhea |
| 17 | HNKF2023-1794 | Male | 55 | General Surgery Department | With Diarrhea |
| 18 | HNKF2023-1803 | Male | 59 | Gastroenterology Department | With Diarrhea |
| 19 | HNKF2023-1810 | Female | 58 | Oncology Department | With Diarrhea |
| 20 | HNKF2023-1811 | Male | 52 | Pain Department | With Diarrhea |
| 21 | HNKF2023-1825 | Female | 62 | Gastroenterology Department | With Diarrhea |
| 22 | HNKF2023-1871 | Female | 55 | Gastroenterology Department | With Diarrhea |
| 23 | HNKF2023-1885 | Male | 62 | Gastroenterology Department | With Diarrhea |
| 24 | HNKF2023-1901 | Female | 60 | Gastroenterology Department | With Diarrhea |
| 25 | HNKF2023-1919 | Female | 35 | Gastroenterology Department | Without Diarrhea |
| 26 | HNKF2023-1938 | Male | 15 | Gastroenterology Department | Without Diarrhea |
| 27 | HNKF2023-1995 | Male | 58 | Gastroenterology Department | With Diarrhea |
| 28 | HNKF2023-2024 | Female | 58 | Oncology Department | Not Recorded |
| 29 | HNKF2023-2114 | Female | 52 | Gastroenterology Department | With Diarrhea |
| 30 | HNKF2023-2118 | Female | 44 | Gastroenterology Department | With Diarrhea |

**Table S4 Concentration determination of purified crRNA.**

| Name | Nucleic acid concentration (ng/μL) | A260/280 | A260/230 |
| --- | --- | --- | --- |
| crRNA-*Cytb* | 1561.353 | 2.082 | 2.418 |
| crRNA-*Cox*3 | 1570.059 | 2.072 | 2.404 |
